# New Appliances and Things Medical

**Published:** 1901-08-17

**Authors:** 


### NEW APPLIANCES AND THINGS MEDICAL.

[We shall be glad to receive, at our Office, 28 & 29 Southampton Street Strand, London, W.C., from the manufacturers, specimens of all new preparations and appliances which may be brought out from time to time.]

### PRIDEAUX'S PURE MILK CASEIN.

(Prideaux's Pure Casein and Life Food Co., Ltd.)
Motcambe, Dorset.

By a special patented process the casein of cow's milk is prepared in a fine flocculent form, dried, and supplied in boxes. This casein is neutral in reaction and is soluble in hot water, affording an opalescent glutinous solution. It is practically free from fat, and contains neither starch nor sugar. For invalids and growing infants this form of food appears specially indicated, and is a great improvement on the majority of patent foods, which consist chiefly of starch or sugar, varieties of food which are by no means suitable for invalids, especially in those cases in which there is gastric disturbance. Starch or sugar in such cases readily undergoes fermentation in the stomach, whereas a proteid food, such as casein, cannot undergo this distinctive process. Further than this, casein, in the form presented by Prideaux's pure milk casein, is exceedingly bland and unirritating to the mucous membrane; it is very easily digested and assimilated. It may be used in a great variety of ways, but perhaps for invalid use it is best employed when added to beef-tea or soup. When mixed with flour and used for ordinary domestic purposes, such, for instance, as for making pastry, cakes, or bread, the nutritive value of these is greatly increased.

